# Physicochemical and biological ageing processes of (micro)plastics in the environment: a multi-tiered study on polyethylene

**DOI:** 10.1007/s11356-022-22599-4

**Published:** 2022-08-22

**Authors:** Gilberto Binda, Giorgio Zanetti, Arianna Bellasi, Davide Spanu, Ginevra Boldrocchi, Roberta Bettinetti, Andrea Pozzi, Luca Nizzetto

**Affiliations:** 1 Norwegian Institute for Water Research (NIVA), Økernveien 94, 0579 Oslo, Norway; 2 Department of Science and High Technology, University of Insubria, Via Valleggio 11, 22100 Como, Italy; 3 Department of Human and Innovation for the Territory, University of Insubria, Via Valleggio 11, 22100 Como, Italy; 4 RECETOX, Masarik University, Kamenice 753/5, 625 00, Brno, Czech Republic

**Keywords:** Pollution, Water chemistry, UV radiation, Biofouling, Microplastics

## Abstract

Pollution by plastic and microplastic impacts the environment globally. Knowledge on the ageing mechanisms of plastics in natural settings is needed to understand their environmental fate and their reactivity in the ecosystems. Accordingly, the study of ageing processes is gaining focus in the context of the environmental sciences. However, laboratory-based experimental research has typically assessed individual ageing processes, limiting environmental applicability. In this study, we propose a multi-tiered approach to study the environmental ageing of polyethylene plastic fragments focusing on the combined assessment of physical and biological processes in sequence. The ageing protocol included ultraviolet irradiation in air and in a range of water solutions, followed by a biofouling test. Changes in surface characteristics were assessed by Fourier transform infrared spectroscopy, scanning electron microscopy, and water contact angle. UV radiation both in air and water caused a significant increase in the density of oxidized groups (i.e., hydroxyl and carbonyl) on the plastic surface, whereby water solution chemistry influenced the process both by modulating surface oxidation and morphology. Biofouling, too, was a strong determinant of surface alterations, regardless of the prior irradiation treatments. All biofouled samples present (i) specific infrared bands of new surface functional groups (e.g., amides and polysaccharides), (ii) a further increase in hydroxyl and carbonyl groups, (iii) the diffuse presence of algal biofilm on the plastic surface, and (iv) a significant decrease in surface hydrophobicity. This suggests that biological-driven alterations are not affected by the level of physicochemical ageing and may represent, in real settings, the main driver of alteration of both weathered and pristine plastics. This work highlights the potentially pivotal role of biofouling as the main process of plastic ageing, providing useful technical insights for future experimental works. These results also confirm that a multi-tiered laboratory approach permits a realistic simulation of plastic environmental ageing in controlled conditions.

**Supplementary Information:**

The online version contains supplementary material available at 10.1007/s11356-022-22599-4.

## Introduction

Plastic litter and microplastic pollution is a global scale concern (Hurley and Nizzetto 2018; Cutroneo et al. 2020; Bellasi et al. 2020). Continuous increase in production, use, and volume of mismanaged plastic waste is mirrored by the increasing accumulation of these materials in the environment (Barnes et al. 2009; Nizzetto et al. 2016; Schell et al. 2021), with water ecosystems acting as final sinks (Schwarz et al. 2019; Bellasi et al. 2020; Binda et al. 2021a).

Knowledge about the environmental behavior of (micro)plastics is crucial to assess the ecological risk (Bond et al. 2018; Min et al. 2020). Understanding environmental behavior requires to elucidate how these materials change in response to physicochemical and biological ageing processes taking place in the environment (Liu et al. 2021). Ultraviolet (UV) and visible light exposure, mechanical stress, and thermo-oxidative processes are known to change the surface roughness, enhance oxidation of surface groups, and cause embrittlement of plastic materials (Luo et al. 2020, 2022; Alimi et al. 2022). These processes, in turn, induce the fragmentation and detachment of smaller particles (Chen et al. 2021), cause the leaching of (toxic) additives (Capolupo et al. 2020; Meng et al. 2021; Allan et al. 2022), and increase the adsorption of a variety of non-polar compounds (Velez et al. 2018; Mosca Angelucci and Tomei 2020). However, UV radiation can be affected in an environmental context by the water’s chemical features. For example, free reactive radicals can be sourced in oxidizing environments (Rosenfeldt and Linden 2007), enhancing the braking of the polymer chain. On the other hand, other water compounds (e.g., dissolved organic matter) can quench UV radiation (Häder et al. 2015) and consequently reduce the surface oxidation of plastic (Chen et al. 2021). Moreover, plastic can likely interact with other chemical stressors during its use life and after its dispersion, especially in urbanized contexts (e.g., in wastewater or sludge treatments). The abovementioned processes, which can further enhance plastic chemical degradation (e.g., alkaline hydrolysis and advanced oxidation processes; Miranda et al. 2021; Alimi et al. 2022), are generally overlooked in laboratory-based ageing tests.

The colonization by bacteria and other microorganisms in water environments is another (poorly understood) driver of (micro)plastic ageing in water and soils (Hurley and Nizzetto 2018). This phenomenon can reshape the physicochemical properties of (micro)plastic, inducing surface oxidation, reducing its hydrophobicity (Chamas et al. 2020), altering surface characteristics, and increasing the bulk density of fragments (Miao et al. 2021a; Bellasi et al. 2021). These phenomena can, in turn, change the environmental fate of (micro)plastic particles (Rummel et al. 2017; Leiser et al. 2020; Duan et al. 2021; He et al. 2021) and can make polymers more likely to adsorb polar compounds, including toxic metals (Mosca Angelucci and Tomei 2020; Binda et al. 2021b).

These various environmental ageing processes have been typically studied individually in laboratory experiments. Although resolving complexity in its constituents is often a good research approach, a “one-at-a-time” strategy may not be appropriate to unfold the relevance of different processes and enable a holistic understanding of the phenomenon. Therefore, experimental setups considering more environmental factors are needed to rank the different ageing processes and their effects on plastic physicochemical properties.

On the other hand, the reconstruction of ageing processes through field-scale experiments (e.g., mesocosms) can present an excessive complexity instead, blurring the information on specific effects induced by different ageing processes. Moreover, the simulation of plastic ageing in natural environments requires long-term, time-consuming experiments due to the notable resistance of plastic polymers. Multi-tiered, laboratory-based experiments at an increasing level of system complexity represent instead the way forward in this research (Binda et al. 2021b; Alimi et al. 2022).

Therefore, in this work, we propose a multi-factorial, laboratory-based investigation of the role of water chemistry, UV radiation, and biofouling in affecting the surface properties of plastic. In this way, the different drivers of plastic environmental ageing are tested in laboratory conditions, avoiding uncontrollable factors typical of field experiments. This type of approach can shed light on the more influencing factors in altering plastic properties after their dispersion, suggesting the next steps towards the creation of reference materials simulating environmentally aged plastic. Polyethylene (PE) was selected as a model polymer considering its wide abundance in all environments (Danso et al. 2019).

## Materials and methods

### Reagents and solutions

All solutions were prepared using ultrapure water (18.2 MΩ resistivity, obtained with a Sartorius Arium mini instrument, Germany). Nitric acid 0.1 M solution was obtained by diluting ultrapure HNO_3_ (65% wt.), obtained by sub-boiling distillation in a Milestone (USA) duoPUR distillation system (Monticelli et al. 2019), while NaOH 0.1 M solution was obtained from 50% in weight NaOH solution (Carlo Erba reagents, Italy). Lake water was obtained from a sampling campaign in Como lake (45°48′55.08’’ N, 9°04′32.1’’ E). Water was filtered with cellulose 0.45 µm pore size filters on site and stored in glass bottles. Physicochemical parameters and major ions composition of the water sample are analyzed following QA/QC protocols already listed elsewhere (Binda et al. 2022), and water chemical features are reported in Supplementary Table S1.

### PE samples preparation and experimental setup

**Fig. 1:**
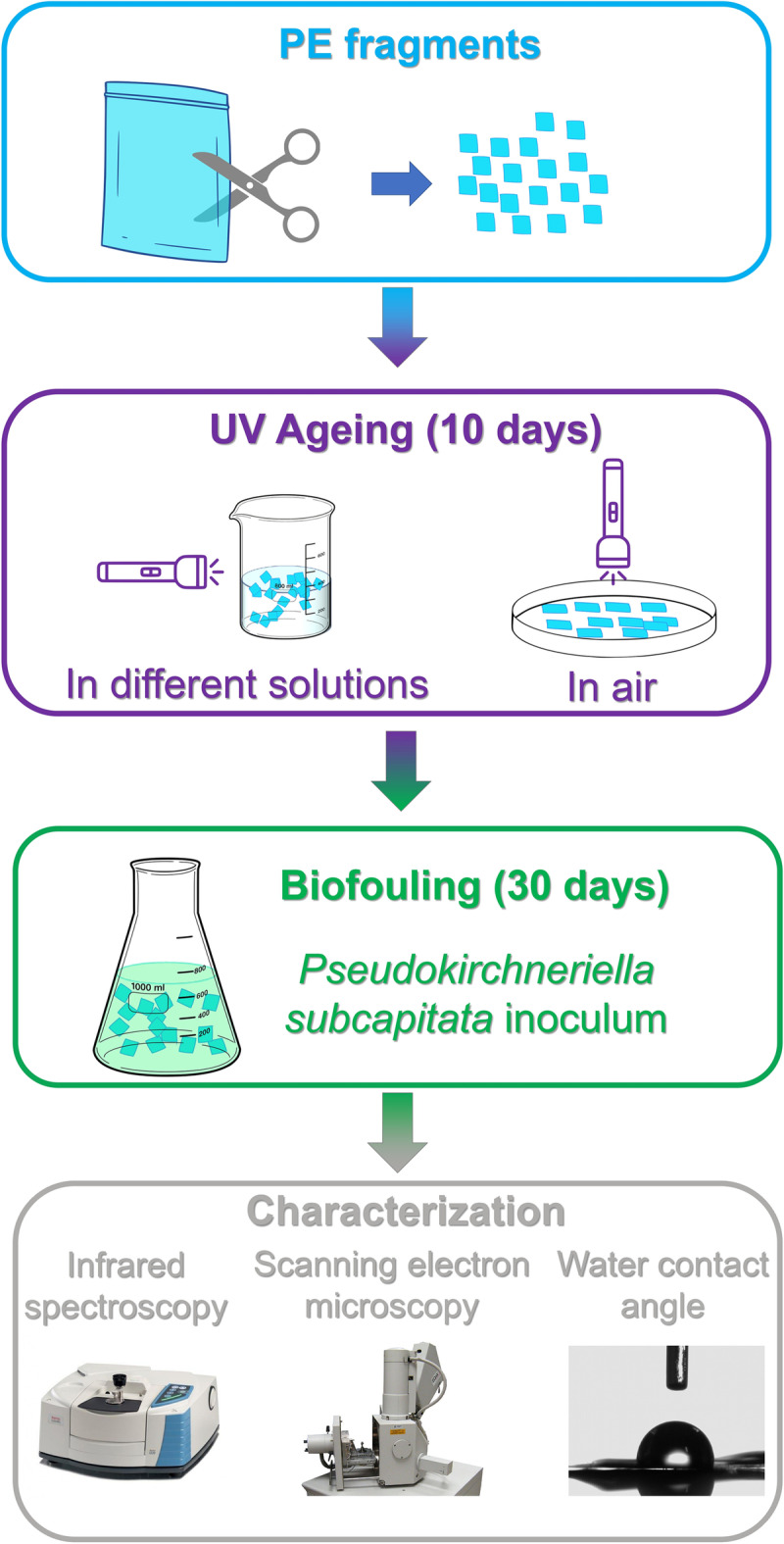
Workflow of the experimental setup

Polyethylene fragments were prepared by cutting a commercial polyethylene transparent bag into squares with a side length of about 4 mm using scissors, reaching a final dimension of the fragments of 4 × 4 × 0.04 mm (in order to potentially simulate macroscopic objects and large microplastic fragments). Then, the following experimental approach was applied (Fig. 1): Samples were exposed to UV radiation in different solutions or in the air for 10 days (see “Physicochemical ageing experiment”); then, part of the aged samples and pristine materials underwent an incubation in axenic microalgal culture (*Pseudokirchneriella subcatipitata*) for 30 days (see “Incubation of plastic fragments”). The biofouling process was not performed simultaneously under UV radiation due to the harsh physicochemical conditions, negatively affecting algae growth (Rastogi et al. 2020).

Finally, all the UV-aged and biofouled samples were analyzed with different analytical techniques to understand changes in surface morphology (with scanning electron microscopy, SEM), reactive surface groups (with Fourier transform infrared spectroscopy, FT-IR), and wettability (through water contact angle measurements). As a control, pristine PE fragments kept in the dark were analyzed too (labeled as pristine in the following sections).

### Physicochemical ageing experiment

Five different ageing media were tested under UV radiation to simulate different environmental stressors:

- 0.1 M HNO_3_ in deionized water (pH ca. 1, sample labeled as HNO_3_), simulating acidic environments in wastewater conditions (Wang et al. 2021) or long-term effects of weaker acidic conditions (e.g., prolonged exposure to acid rains, Miranda et al. 2021);
- 0.1 M NaOH in deionized water (pH ca. 13, sample labeled as NaOH), to simulate a strongly alkaline environment and enhance alkaline hydrolysis of the polymer, simulating long-term degradation in weaker alkaline conditions (Miranda et al. 2021). This process can also simulate sludge dewatering processes, likely affecting plastic fragments (Liu et al. 2022);
- 33% H_2_O_2_ (sample labeled as H_2_O_2_), a liquid medium used to simulate thermo-oxidative degradation of plastic (Luo et al. 2021; Bhagat et al. 2022). This process can also simulate the effects of advanced oxidation processes (Alimi et al. 2022; Liu et al. 2022), likely to affect plastic particles after their dispersion (e.g., in wastewater treatment plants);
- lake water medium, to simulate a natural freshwater environment (sample labeled as LW). Freshwater was selected because of the emerging concern on the impacts of microplastics in soils and freshwater systems, less studied than seawater environments (Bellasi et al. 2020);
- air, to observe the UV-derived ageing only, excluding the effect of water media (sample labeled as air).

To simulate the light degradation in different aquatic media, ageing experiments were conducted in a quartz vial, under magnetic stirring, and irradiating the sample with UV radiation perpendicular to the vial (a video of the system during ageing experiments is presented in Supplementary Video S1). An LED UV-A light source (Alonefire SV13 15 W, China, with a dominant wavelength of 365 nm) was used with a radiation intensity of approximately 1 mW cm^−2^, as measured using a thermal sensor-based power meter (OptoSigma, France). These conditions were tested in accordance with the abundant body of literature applying UV-A radiation as a sunlight simulation for plastic ageing (Liu et al. 2021). The shaking speed was set at 150 rpm, and the temperature was kept at 25 ± 2 ℃ (Chen et al. 2021). Plastic specimens were collected after 10 days of ageing: After this time, the total dose of UV-A radiation is comparable to 50 days of continuous natural sunlight with a clear sky in a temperate climate region (Pieristè et al. 2019). This UV radiation dose is observed to be sufficient in inducing surface oxidation on plastic (Martínez et al. 2021; Sarkar et al. 2021; Bhagat et al. 2022). After the ageing process, plastic fragments were filtered on a 0.45 µm pore size cellulose filter, rinsed with ultrapure water, and left to dry for 24 h in air. The fragments aged in the air were instead placed in a petri dish and exposed to UV radiation for the same time period and at the same radiation intensity (i.e., 10 days at approximately 1 mW cm^−2^ of radiation intensity).

### Incubation of plastic fragments

After the physicochemical ageing tests in different media, fragments were exposed to the algal culture to simulate biofouling in freshwater environments. Pristine PE fragments were also incubated to test if the previous plastic degradation can alter the rate of biofouling development. Moreover, a batch with plastic fragments without algal culture (sample named Control-B) was prepared to check possible photoinduced degradation during the incubation experiment.

Briefly, 80 mg of each type of plastic fragment was added in glass Erlenmeyer flasks containing 50 mL of growth water medium, prepared according to OECD guidelines (OECD 1984). Then, about 0.5 mL of *Pseudokirchneriella subcapitata* inoculum was added, obtaining a starting cell density of 68,000 cells/mL. This green alga was selected as a model organism to represent the mature biofilm observed on (micro)plastics in photic environments, where algae represent an important component of plastic biofilms (Arias-Andres et al. 2018; Di Pippo et al. 2020; Smith et al. 2021; Nava et al. 2021). Moreover, from the practical point of view, this algal species was selected for its abundant use in laboratory experiments (mostly as model organisms for ecotoxicological tests, Ceschin et al. 2021) and its consequent availability.

All the flasks were exposed to continuous visible light (400–700 nm range, intensity 0.3 mW/cm^2^) under shaking. Cell growth was measured after 3 days, 7 days, 14 days, and 30 days in all the flasks through visual sorting using a Burker counting chamber and Zeiss (Germany) PrimoStar optical microscope (measurements were performed in triplicates). Plastic fragments were finally collected after 30 days of incubation to permit to the suspended culture to reach the growth climax and achieve stable biofouling (Amaral-Zettler et al. 2021; Kiki et al. 2022). Then, the solution was filtered to extract PE fragments (as made for UV-aged fragments), which were then gently rinsed with ultrapure water in order to remove loosely attached organisms, and finally left for 24 h in the air to dry (Xiong et al. 2019). Biofouled fragments were then stored for surface properties analysis without removing the attached algae to observe how the priming and colonization of plastic fragments affect the surface properties. All the biofouled samples will then be presented in results using the label of the previous UV treatment (HNO_3_, NaOH, H_2_O_2_, LW, and air, respectively), adding -B as an ending, while the pristine-B label will be used for the biofouled pristine PE specimen.

### Surface characterization

The obtained dried PE fragments were characterized for their morphology and surface functional groups. The micromorphology of plastic samples was investigated using a Philips® (the Netherlands) Field Emission Gun-Scanning Electron Microscope (FEG-SEM), with a 20 keV beam under high vacuum conditions. Elemental analysis on the surface was performed with an energy dispersive X-ray probe (EDX). To make the plastic surface more conductive, samples were uniformly covered with a ca. 5 nm thick gold layer using a Cressington (United Kingdom) 108 auto vacuum sputter coater before SEM analysis. Biofouled specimens were also analyzed using a Nikon (Japan) SMZ 745 T stereomicroscope to observe the distribution of algal biofilms on plastic fragments.

To investigate the changes in surface functional groups of PE fragments, attenuated total reflectance infrared spectroscopy (ATR-FT-IR) was performed using a Thermo Scientific™ Nicolet™ iS™ 10 FT-IR Spectrometer (USA) on pristine and aged plastics; 32 scans for each sample were performed in the spectral range 4000–650 cm^−1^, with a resolution of 0.482 cm^−1^. Before the spectral analysis of every sample, a background spectrum was collected.

The water contact angle was tested through a drop shape analyzer (OCA-20, Dataphysics, Germany) by using the sessile drop method. Approximately 2 μL of distilled water was dropped on the surface of the plastic sample through a syringe; then, the contact angles were computed with SCA20 software.

All surface analysis techniques were performed on three replicate plastic specimens: SEM micrographs present high similarities among samples, while FT-IR spectra and water contact angles present a relative standard deviation of 5% (absorbances values) and 7% (degrees), respectively.

### Spectral data analysis

Before further treatments, all FT-IR data were smoothed using Savitzky–Golay filter (30 points of the window) and scaled on the maximum absorbance peak. The different FT-IR peaks were then checked using the “peak analyzer” function of Origin 2018 software (OriginLab Corporation).

Then, the carbonyl index was calculated as the ratio of the C = O peak at 1715 and the C-H stretching at 1465 (Martínez et al. 2021), while the hydroxyl index was obtained from the ratio of the maximum absorbance in the window 3600 to 3100 cm^−1^ and the absorbance of the C-H stretching at 1472 cm^−1^ (Yang et al. 2021). These indexes were calculated to obtain a quantitative reference of surface oxidation of polymers (after UV ageing) as well as the presence of these functional groups in the biofouled fragments. These indexes were also compared with the FT-IR spectra of 4 environmental samples of plastic litter (composed by PE) collected on a lake shore (Bellasi et al. 2022).

A *t*-test was performed on these data using Origin 2018 software (OriginLab Corporation 2018) to observe statistically significant differences among the different treatments.

To further assess the differential changes in FT-IR spectra during the different tested ageing processes, principal component analysis (PCA) was applied to the spectral data. This multivariate statistical tool was widely applied to FT-IR data to extract the main trends in spectral changes (Gurbanov et al. 2018; Cavaglia et al. 2020; Gorla et al. 2020) and was recently applied also to understand the UV ageing of different polymers (Zvekic et al. 2022). Specific spectral windows showing major changes after ageing were selected prior to the analysis (namely, 3600–3000 cm^−1^, 1800–1500 cm^−1^, and 1400–800 cm^−1^; see “Physicochemical ageing: the effects of water chemistry” and “The role of biofouling and potential environmental impacts”). PCA was computed using R software (R Core Team 2014).

## Results and discussion

### Physicochemical ageing: the effects of water chemistry

**Fig. 2:**
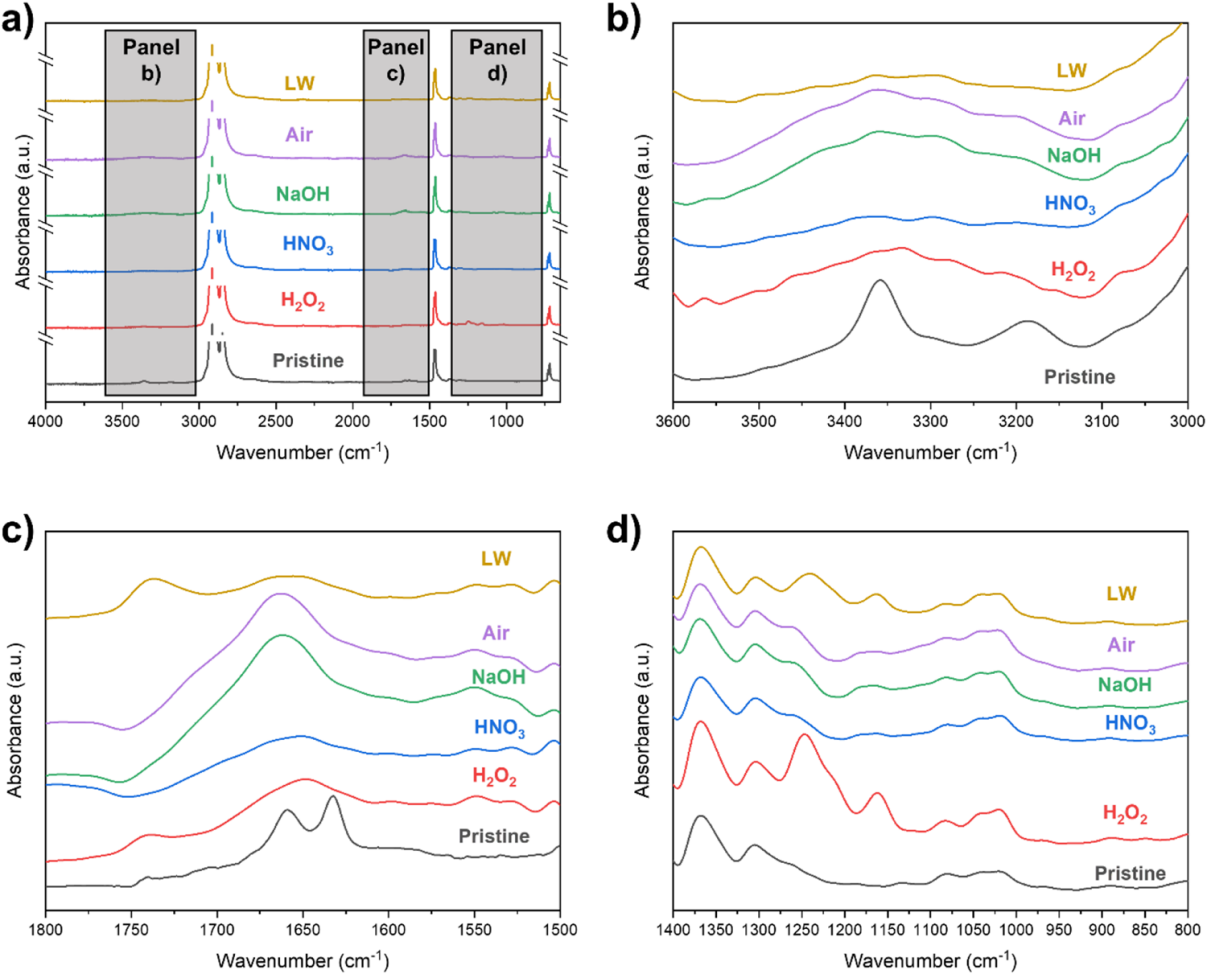
FT-IR spectra of the differently treated PE plastic fragments in the different media: lake water (LW, in brown); air (in violet); NaOH (in green); HNO_3_ (in blue); H_2_O_2_ (in red). The control Pristine PE is also shown (in black). Figure (**a**) shows the whole spectral window (the peaks at 2822 cm^−1^ and 2810 cm^−1^ are cut for the sake of clarity), and the three gray panels indicate the specific spectral windows analyzed at 3600–3000 cm^−1^ (**b**), 1800–1500 cm^−1^ (**c**), and 1400–800 cm.^−1^ (**d**)

The effect of 10-day UV ageing on PE specimens generally shows that changes are mostly observable in regions of the spectrum representing oxygenated surface groups (Fig. 2a): hydroxyl groups (at 3600–3000 cm^−1^), carbonyl groups (at 1800–1500 cm^−1^), and esters and vinyl groups (at 1400–800 cm^−1^), in accordance with previous reports (Song et al. 2017; Fairbrother et al. 2019; Kalčíková et al. 2020; Chaudhary and Vijayakumar 2020).

Observing the 3600–3000 cm^−1^ window, a broad band of alcoholic -OH was clearly visible in UV-aged plastic in air, as well as for specimens aged in alkaline and oxidizing conditions (NaOH and H_2_O_2_ samples in Fig. 2b). This is possibly ascribed to a higher concentration of •OH radicals in water derived from the alkaline conditions (Huang et al. 2008) and in the presence of H_2_O_2_ under UV radiation (Liu et al. 2019): These radicals are known to induce the formation of surface -OH groups on various polymers (Ross et al. 2000; Zha et al. 2021). All the other fragments aged in water solutions presented spectra with a less marked -OH peak compared to the sample aged in air. Water is known to inhibit surface oxidation induced by UV radiation, absorbing UV radiation and reducing the photoinduced photolytic cleavage of C-H bonds in polymer backbone (Gewert et al. 2015). Moreover, all the aged samples showed the loss of two peaks at 3350 cm^−1^ and 3200 cm^−1^ (indicating N–H stretching in amides, Parker 1971).

**Fig. 3:**
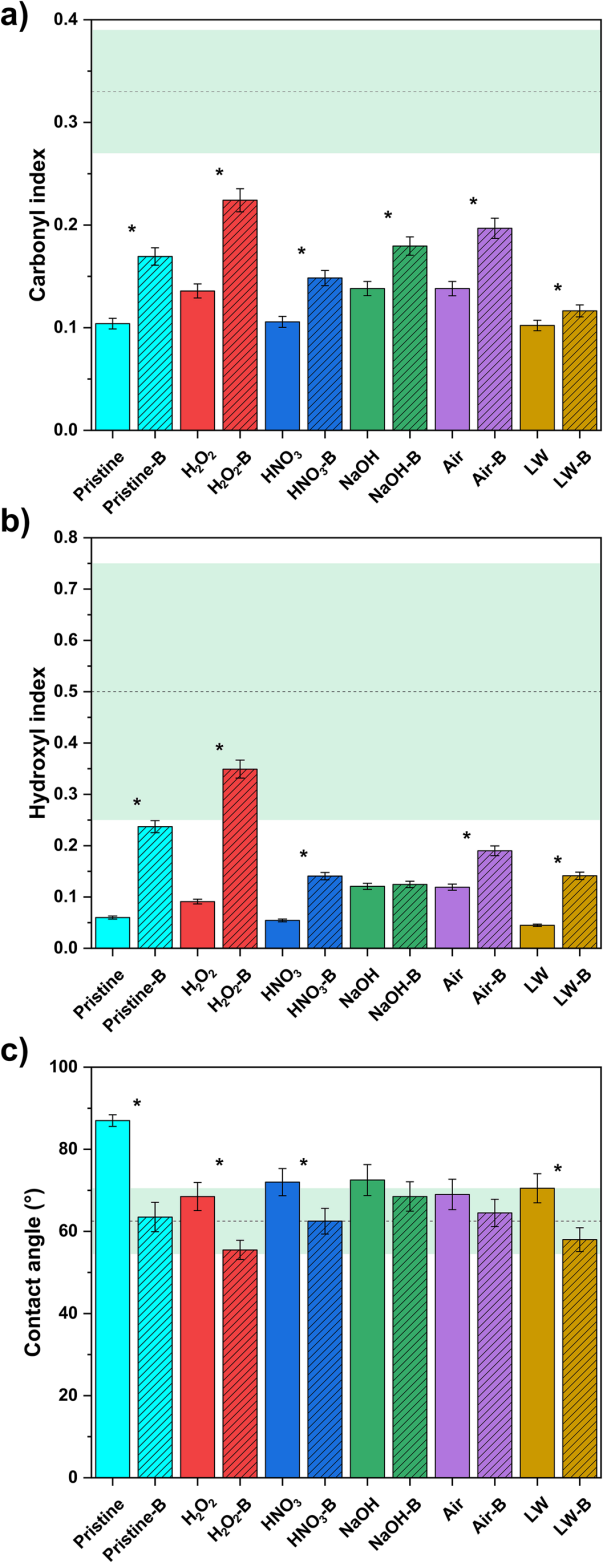
Bar plot of carbonyl index (**a**), hydroxyl index (**b**), and water contact angle (**c**) of pristine and UV-aged plastic polymers before (solid fill) and after biofouling (striped pattern). Bars are color-indexed by the different physicochemical ageing processes, and error bars indicate the standard deviation (after three replicates). Significantly different values after biofouling (*p* < 0.05 after *t*-test) are indicated with an asterisk, while the light green dashed area indicates the average ± standard deviation range of environmental samples (Bellasi et al. 2022)

In the 1800–1500 cm^−1^ range, a reshaping of the peaks is observable for almost all the treatments, with more marked changes in air and NaOH treatment. A broad band of the stretching vibrations between non-conjugated C = C bonds at 1680 cm^−1^ is observable, in accordance with other field studies on PE (Abed et al. 2020). Unlike previous reports (Liu et al. 2019), C = O peaks of carboxylic acids and ketones at 1730 cm^−1^ are observable in a few samples analyzed in this study (LW and H_2_O_2_, Fig. 2c). For the LW sample, the peak at 1730 cm^−1^ is possibly ascribable also to the priming of dissolved organic matter, as a conditioning film on the PE fragments (Rummel et al. 2021). The limited abundance of carbonyl functional groups on the plastic surface after UV ageing is further confirmed by the values of carbonyl index, which results lower than biofouled and environmental samples (Fig. 3a).

Comparing then the aged samples with the pristine spectrum, the loss of two peaks at 1660 cm^−1^ and 1630 cm^−1^ (representing the acyclic C = C bond and C = O bond in amides, respectively, Fig. 2c) is observable in all the treatments. These observations, in addition to the loss of the other two peaks in the 3500–3100 cm^−1^ window (Fig. 2b), highlight the leaching in the water of an amide-containing compound after the initial ageing of the plastic material. Amide-containing compounds are, in fact, often added as slip agents in polyethylene (Nielson 1991).

In the 1300–1100 cm^−1^ window, instead, only H_2_O_2_ and LW specimens show some alterations in functional groups after UV ageing (Fig. 2d). In the former, the oxidative effect of H_2_O_2_ is observable with two evident peaks at 1250 cm^−1^ and 1150 cm^−1^ of C-O stretching, as an index of ester formation (Fotopoulou and Karapanagioti 2012). These peaks are present but less marked in the LW sample.

The marked surface oxidation of plastic also expectedly induced a decrease in hydrophobicity of its surface, as observed by the decrease of water contact angle in all UV treatments compared to pristine plastic (Fig. 3c). This observation is in good accordance with previous studies testing the wettability of UV-aged plastic (Chen et al. 2021).

**Fig. 4:**
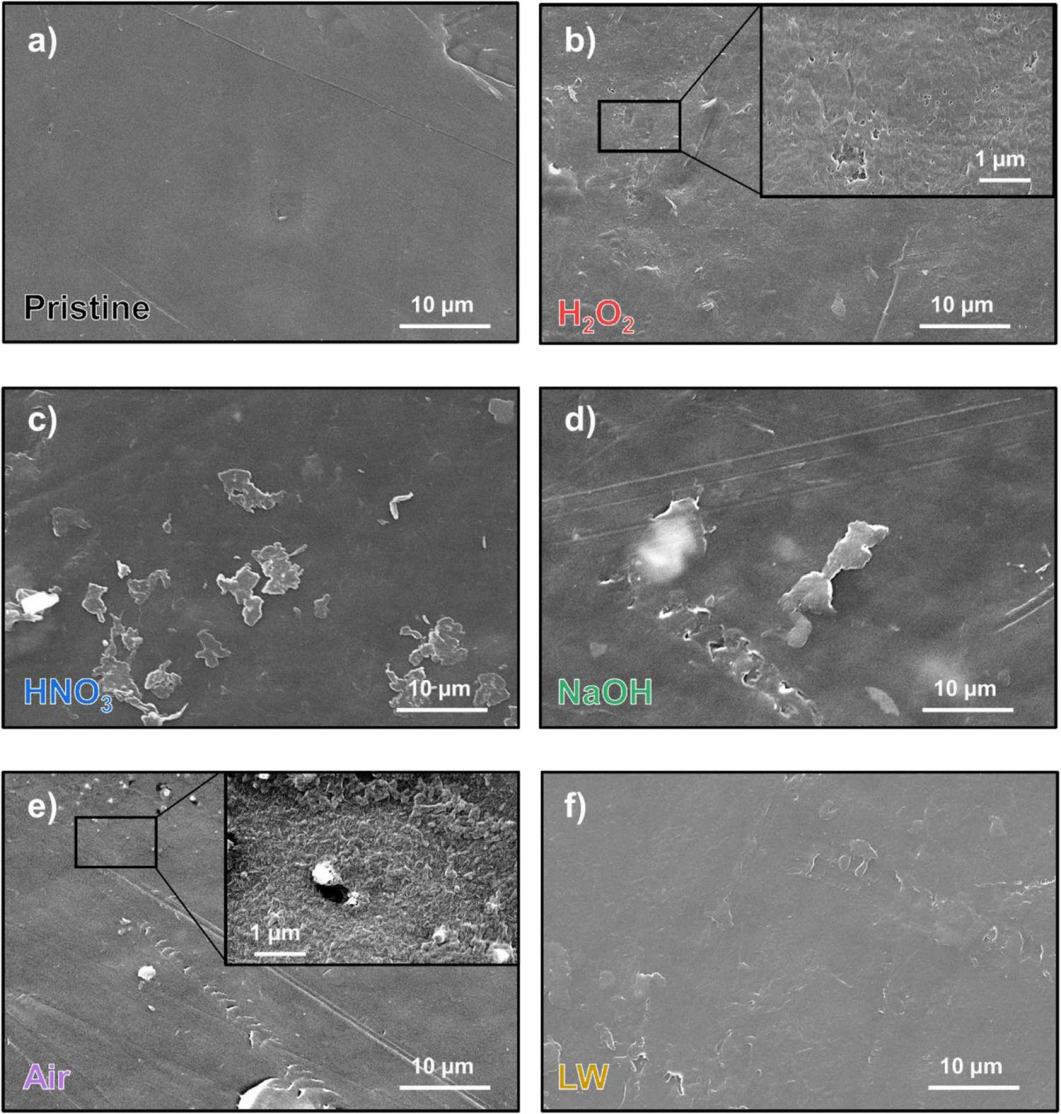
SEM micrographs of the different PE specimens at 2000 × magnifications: (**a**) pristine PE; samples UV-aged in H_2_O_2_ (**b**), HNO_3_ (**c**), NaOH (**d**), air (**e**), and lake water (**f**). A detail at 16,000 × magnifications is presented in (**b**) and (**e**)

Beyond the formation of oxygen-containing surface functional groups, UV ageing leads to changes in plastic surface morphology and to the embrittlement of polymer structure (Fig. 4), in accordance with other observations in literature (Luo et al. 2021). This issue seems to be mostly related with surface oxidation, but our results highlighted that this is also affected by the pH of ageing media. As observable from SEM images, both an acid (HNO_3_, Fig. 4c) and an alkaline environment (NaOH, Fig. 4d) change the pattern of surface degradation. Abundant fragmentation of the plastic samples is observable, with fragments in the order of 1–10 µm detaching from the plastic surface. The strong surface degradation is in accordance with the abundant oxidation observed from FT-IR spectra of NaOH-aged specimens (Fig. 2), confirming increased embrittlement of the polymer structure. In contrast, this is unexpected for the HNO_3_ treatment since plastics treated with this reagent showed limited oxidation in the FT-IR spectra (Fig. 2). This behavior is possibly due to the embrittlement of PE induced by acidic conditions, regardless of the surface oxidation (Geburtig et al. 2018). Acidic conditions, in fact, are also observed to negatively affect the tensile strength of PE films (Wang et al. 2021).

UV treatment in air and the treatment with H_2_O_2_ led to the formation of small holes (in the order of 100 nm) on the polymer surface, which can be the initiation sites of oxidative radical-induced chain reactions in the amorphous phase of the polymer matrix (Pickett 2018).

As previously observed, the presence of dissolved organic matter, other dissolved ions, and water itself can instead inhibit the effects of UV radiation on surface alteration (Gewert et al. 2015; Wang et al. 2021). Incidentally, surface degradation was less marked in specimens exposed to UV radiation in lake water (Fig. 4f).

### The role of biofouling and potential environmental impacts

After 30 days of biofouling experiments, the algal populations in the batches are at their maximum growth, with a population density between 3.2 × 10^7^ and 3.9 × 10^7^ cells/mL (see Supplementary Fig. S1). No significant differences in algal growth were observed among the batches. All the batches presented PE specimens covered by algae on a macroscopic scale (Supplementary Fig. S2).

**Fig. 5:**
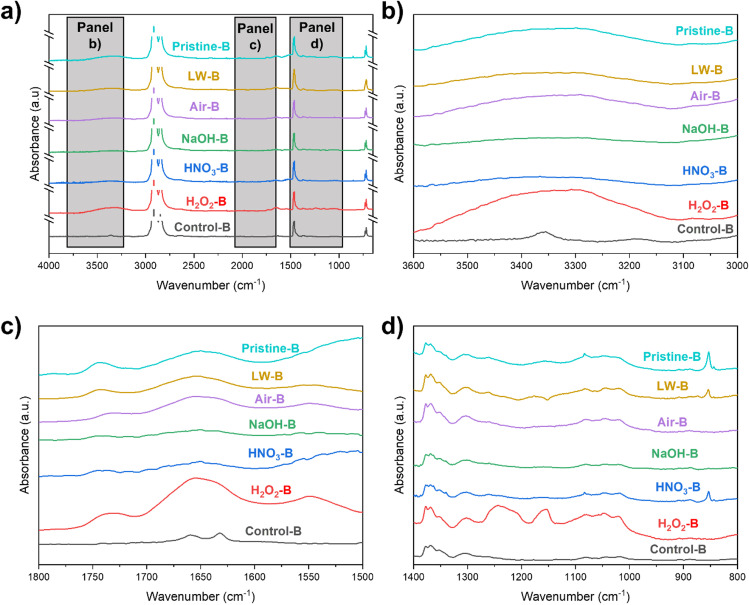
FT-IR spectra of the biofouled PE without pre-treatment (pristine-B, in light blue) and after the different ageing processes in lake water (LW-B, in brown), air (air-B, in violet), NaOH (NaOH-B, in green), HNO_3_ (HNO_3_-B, in blue), and H_2_O_2_ (H_2_O_2_-B, in red). The control-B specimen is also shown (in black), suggesting that the visible light used for algal growth poorly affects plastic surface groups after 30 days of incubation. Figure (**a**) shows the whole spectral window (the peaks at 2822 cm^−1^ and 2810 cm^−1^ are cut for the sake of clarity), and the three gray panels indicate the specific spectral windows analyzed at 3600–3000 cm^−1^ (**b**), 1800–1500 cm^−1^ (**c**), and 1400–800 cm.^−1^ (**d**)

Whole FT-IR spectra and specific spectral windows of biofouled specimens are observable in Fig. 5. Differences are observable compared to the specimens that only underwent physicochemical ageing, especially in specific the spectral windows of hydroxyl, carbonyl, and vinyl groups (Fig. 5b, c, d). Starting from the -OH region at 3600–3000 cm^−1^, the patterns result mostly similar to the UV-aged specimens (Fig. 2b), but all the biofouled specimens except NaOH-B presented a higher -OH band absorption, as observable by the significantly higher hydroxyl index values, similar to environmentally collected plastic (Fig. 3b). Moreover, pristine PE samples after biofouling presented a wide -OH band too. This can be related to the abundant presence of -OH-containing compounds (e.g., cellulose, hemicellulose, and pectin) at the surface of microalgal biological material (Binda et al. 2020; Madadi et al. 2021).

In the 1800–1500 cm^−1^ window, a re-shaping of the peaks of C = O carbonyls, with a broad peak at 1740 cm^−1^, is observable after biofouling in air-B, H_2_O_2_-B, and LW-B, as well as on pristine-B specimens. This is instead less marked in NaOH-B and HNO_3_-B samples (Fig. 5c). This re-shaping can be caused by the lipids of *Pseudokirchneriella subcapitata*, rich in carbonyl functional groups (Xiong et al. 2020): Their presence also causes a significant increase in carbonyl index values in all the plastic specimens tested, with values closer to environmentally collected plastics in comparison to UV-aged samples (Fig. 3a).

Another explanation evoked for the presence of the increasing FT-IR peak at 1730 cm^−1^ after longer ageing times is the biodegradation of the polymer by the colonizing microorganisms (Tu et al. 2020). This issue, however, can be ruled out in our case since this peak was only sparsely observed in UV-oxidized plastics (Fig. 2c), while UV radiation is normally considered as the main driver of carbonyl formation on polymer surfaces.

Moreover, two new FT-IR peaks are observable in LW-B, air-B, and H_2_O_2_-B samples at 1650 cm^−1^ and 1550 cm^−1^ representing primary and secondary amides, respectively (Rahman et al. 2018). These peaks further confirm the appearance of different surface functional groups once the plastic is covered by microbiota and likely represents the polypeptide structures of algae (Helm and Naumann 1995; Xiong et al. 2020). Moreover, HNO_3_-B and pristine-B samples present a broader band at 1550 cm^−1^, possibly indicating the recombination of the amide with other substituents (Rahman et al. 2018). NaOH-B specimens show instead lower adsorption in these bands compared to other biofouled samples.

Finally, analyzing the 1400–800 cm^−1^ window, most of the patterns observed after UV ageing of samples (including the peaks present only in the H_2_O_2_-aged sample, Figs. 2d and 5d) are unvaried. However, 3 out of 6 samples presented a new sharp peak at 860 cm^−1^ (indicating a glycosidic bond) and a partial reshaping of the peak at 1080 cm^−1^ (C-O stretch of alcohols). This indicates the presence of polysaccharides, derived from the algal colonization of plastic fragments (Kim et al. 2003). This issue was already observed in PE after the incubation with fungi (Chaudhary and Vijayakumar 2020). However, the uneven colonization of algae on plastic samples likely made this peak not present in all the incubated specimens, due to a discontinuous coverage of plastic fragments. This uneven distribution is clearly observable from the pictures of biofouled samples (Supplementary Fig. S2) and causes a heterogenous response in FT-IR bands of functional groups which are not abundant in the biofilms.

**Fig. 6:**
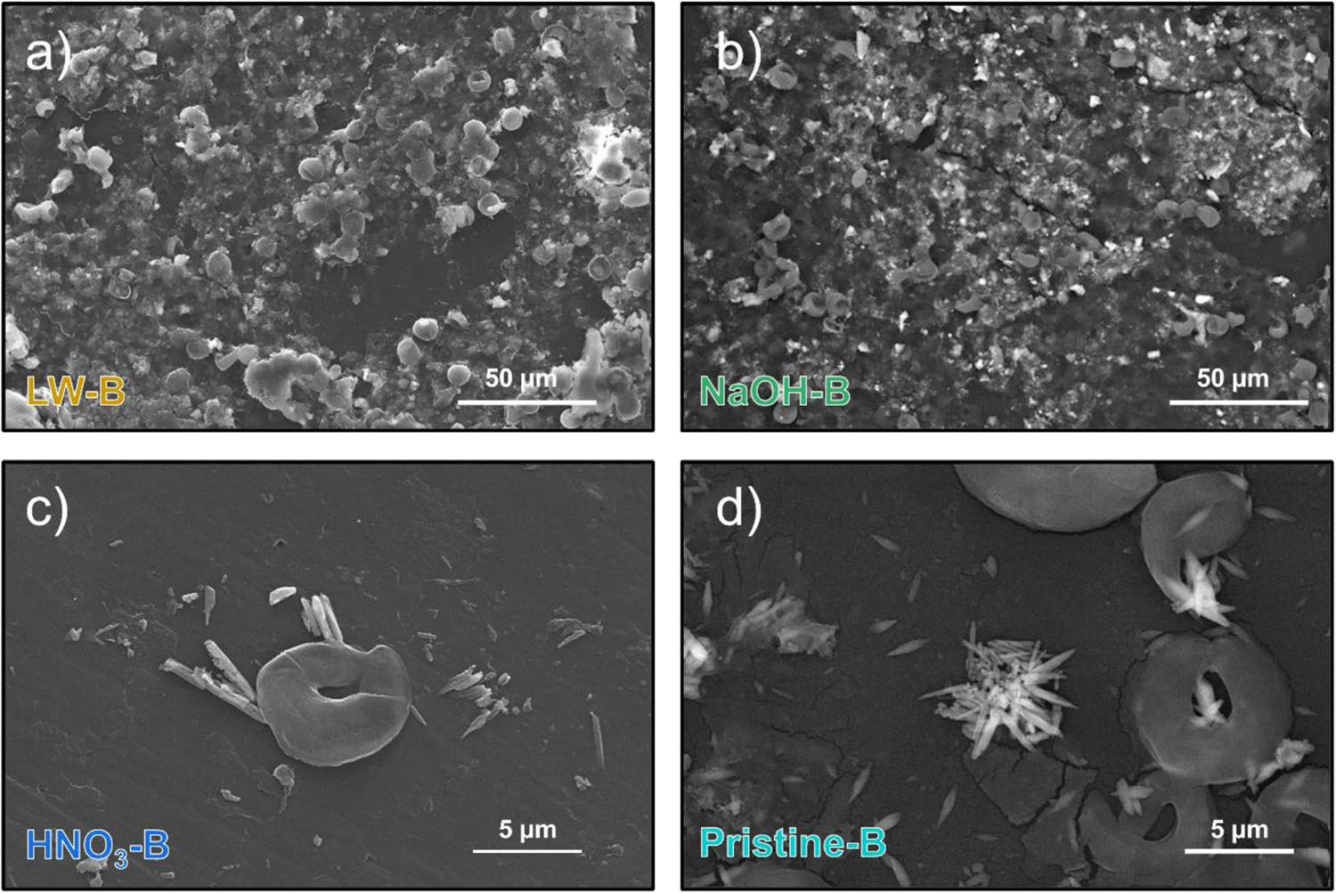
SEM micrographs of samples after biofouling at 500 × magnifications: (**a**) specimens previously UV-aged in lake water (LW-B) and (**b**) specimens previously UV-aged in NaOH (NaOH-B). Details of algal priming on the plastic surface at 4000 × magnifications, with the presence of salty depositions, after ageing in HNO_3_ (HNO_3_-B, panel **c**) and from unaged PE (pristine-B, panel **d**)

The biofilm formation on PE is also reshaping the surface morphology and wettability of plastic, independently from the previous ageing. Observing the morphology of all biofouled specimens, different agglomerates of adhered *Pseudokirchneriella subcapitata* cells are observable in all the samples, regardless of the previous treatment (Fig. 6 and Supplementary Fig. S3).

The adhesion of algae is observed to happen also on the smooth surfaces of plastic, and not only in surface defects (Fig. 6c,d). Moreover, UV-aged and pristine plastic (presenting different wettability, Fig. 3c) show similar biofilm coverage after 30 days (Supplementary Fig. S2). This issue indicates that the changes in micromorphology, the enrichment in oxygen-containing functional groups, and the increased wettability of UV-aged plastic limitedly affect the formation of a stable biofilm on PE fragments.

All the attached microalgae appear on the plastic surface in concomitance with a dense layer observable from SEM images (likely generated by extracellular polymeric substances) and various salt depositions, derived from the accumulation of nutrients from the water by the microbial species. The EDX analyses of these salts indicate the concentration of Ca, K, Na, and Cl up to 25%, 12%, 4%, and 13% in weight, respectively (Supplementary Table S2). This is in accordance with previous studies performed on PET and PLA after incubation in saltwater (Bhagwat et al. 2021), as well as with the elemental analyses of cyanobacteria-based biofilm analyzed on environmentally aged plastics (Leiser et al. 2021). This issue, moreover, presents a potential environmental side effect: Biofilm on plastic, in fact, can actively accumulate elements on the surface of plastic, inducing potential alterations of the natural cycling of elements in water bodies, as well as possibly affecting the environmental fate of potentially toxic elements in polluted environments (Seeley et al. 2020; Binda et al. 2021b).

Another effect of the biofilm covering on the plastic surface is the further increased wettability, as observed by contact angle measurements (Fig. 3c). The further change in contact angle after biofouling is statistically significant for all samples but NaOH-B and Air-B. The increase in wettability due to biofouling was already observed after the incubation in seawater (Tu et al. 2020) and indicates the increased affinity of biofouled (micro)plastic toward the adsorption of polar compounds and metals (Binda et al. 2021b).

### Chemical and biological ageing: comparison of the effects on (micro)plastic properties

The results described above confirm UV radiation to be the main determinant of changes in the plastic specimen properties, increasing oxygen-containing functional groups and enhancing surface roughness (Davranche et al. 2019; Fairbrother et al. 2019; Martínez et al. 2021; Wang et al. 2021). However, here, we show that the chemical condition of the water media can influence the ageing processes and, potentially, also the environmental behavior of (micro)plastics in water bodies. It is worth considering that acid and alkaline environments can enhance physical plastic degradation, possibly facilitating the formation of microplastics in extreme environments such as industrial wastewater pipelines or effluent points.

More importantly, the results highlight an important role of biofouling in modifying plastic surface characteristics, regardless of the previous ageing conditions of the specimens: Similar to irradiated specimens, pristine PE fragments were also colonized by biofilms. After biofouling, all the specimens present similar changes in plastic surface morphology, hydrophobicity, and chemical functional groups, altering the original surface property of plastic (Figs. 3, 5, and 6). As a further confirmation, the abundance of carbonyls and hydroxyl groups and the grade of wettability of biofouled specimens present a higher similarity with environmental samples compared to the UV-aged specimens (Fig. 3).

**Fig.7:**
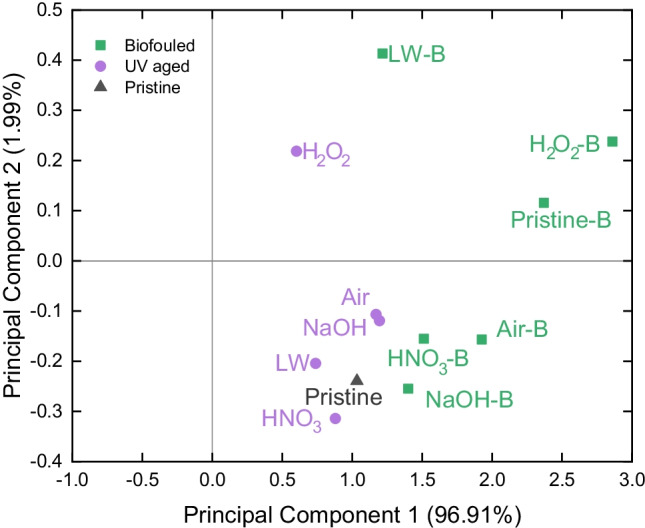
Score plot of principal components 1 and 2 (explained variance is indicated in parenthesis). All biofouled samples are indicated with green squares, UV-aged samples with purple circles, and the pristine sample is indicated with the black triangle

The independence of chemical and biological ageing of (micro)plastics is further highlighted by the scores plot after PCA of FT-IR spectra (Fig. 7): All specimens show a shift with higher values of component 1 (explaining 96.91% of the total variance) after biofouling compared with UV-aged samples. Similarly, the pristine-B sample also shows this shift in comparison with the pristine control sample. This observation confirms that biofouling affects the surface functional groups of all the specimens in a similar way, generating similar bands in all samples (especially hydroxyl groups and amides, which FT-IR bands show high positive loading values, Supplementary Fig. S4). This shift is instead less marked on component 2, where most of the samples with similar chemical ageing present similar score values. Only LW and pristine specimens show instead a major change in component 2 scores after biofouling, possibly related to the higher change in intensity of hydroxyl peak. This peak shows, in fact, the highest loading values on component 2 (Supplementary Fig. S4).

However, similarly to previous biofouling experiments performed in the field (Delacuvellerie et al. 2021), the changes in surface properties are observed to be not homogeneous across the particle surface in the different samples. This is observable, for example, by the appearance of the sharp peak of glycosidic bond in only half of the samples (Fig. 5d), and it is evident from the picture of biofouled plastic fragments in Supplementary Fig. S2. The main explanation for the uneven distribution of biofilms on PE fragments is that the biofouling process generally involves microbial adhesion as a first phase: This is a mostly random process, depending on the type and viability of pioneering organisms possibly anchoring and growing on the substrate (Binda et al. 2021b). Only after this phase, extracellular polymeric substances secretion and microbial proliferation can take place (He et al. 2021; Binda et al. 2021b; Barros and Seena 2021). Our results show anyway that this random process is poorly affected by UV ageing since abundant biofouling is also observed on pristine PE (pristine-B in Fig. 6d and Supplementary Figs. S2a and S3a). Therefore, the role of plastic properties and micromorphology seems to be marginal in the formation of a mature biofilm after 30 days. More detailed investigations are instead needed to understand if the surface properties of plastic or other processes happening in the water environment (e.g., the priming of organic matter) can influence the initial colonization of microorganisms and biofilm growth on plastic (Bhagwat et al. 2021).

### Toward a better understanding of environmental plastic ageing

This study shows that the chemistry of water can alter the surface properties of plastic during ageing by UV, suggesting that plastic polymers can potentially act differently once dispersed in different water and urban environments.

However, an evident role in plastic environmental behavior is indeed played by biofilm formation on plastic fragments. Our multi-tiered laboratory test confirms this hypothesis, showing that plastic surface physicochemical properties are strongly affected by biofouling. Since the colonization of plastic surfaces by different microorganisms has been reported for litter and microplastics collected in a variety of aquatic systems (Nava and Leoni 2021; Delacuvellerie et al. 2021; Deng et al. 2021; Nava et al. 2021), this process needs further investigation in future studies, in order to more realistically mimic the environmental behavior of (micro)plastics.

Studies of plastic ageing in environmental settings showed that biofilm’s structural and functional features are controlled by the environmental conditions and substrate types (Delacuvellerie et al. 2021; Barros and Seena 2021; Nava et al. 2021). However, field experiments still present high variability derived from local conditions and uncontrollable parameters (e.g., climate and complex ecosystem) which can blur the effects of different attaching organisms and of the water conditions in regulating (micro)plastic environmental behavior.

Our analyses on an axenic strain showed that even using a simplified biological model, colonization is efficient to mimic this process in laboratory conditions, in agreement with an earlier study (Bhagwat et al. 2021). Future experiments should fill the gap between simplified laboratory experiments and uncontrollable field ones, focusing on incubation tests with mixed strains containing selected taxa of algae, fungi, and bacteria to understand how different polymers age under different communities with prevailing autotrophs vs. heterotrophs (Miao et al. 2021b). These improvements will possibly lead to the generation of reference materials of naturally aged plastics, for a more realistic simulation of the environmental behavior of aged plastic particles in future experimental studies.

An improved understanding of this process in controlled conditions will help to understand the potential cascade effects on the natural environment, such as changes in plastic environmental fate (Amaral-Zettler et al. 2021), increased adsorption of polar and inorganic compounds (Binda et al. 2021b), and alterations on the cycle of nutrients (Seeley et al. 2020).

## Conclusions

This study combined the assessment of physical, chemical, and biological ageing on plastic fragments in order to reconstruct (micro)plastic ageing in water environments, using PE fragments as reference. The ageing tests were performed in different water solutions under UV radiation, followed by the incubation of plastic fragments in a selected strain of microalgae. The surface analyses highlight that the main processes affecting plastic in the environment are mostly related to UV-induced oxidation, but that also the different water media can enhance or reduce this effect. It is observable that oxidizing acid and alkaline environments determines different formations of surface functional groups and affects plastic rugosity and physical degradation.

This study, moreover, highlights that biofouling affects the surface properties of plastic, regardless of previous treatments, and also affects pristine plastic. Biofouling determines the formation of specific functional groups representing polypeptides and polysaccharides, as well as a strong alteration of microscopic surface structure. Biofouling appeared to proceed independently from the previous level of ageing of the plastic specimen, including pristine PE. Future studies addressing the environmental behavior of plastics and their surface interaction with water chemical constituents and contaminants should consider chemical and biological ageing processes, enabling tracking of the effects of formation and progression of biological colonies on (micro)plastic surfaces. An in-depth understanding of the biofouling process will permit the creation of more realistic aged (micro)plastic reference materials.

## Supplementary Information

Below is the link to the electronic supplementary material.

Supplementary information includes: Tables S1-S2 and Figures S1-S4 (Supplementary file1); raw FT-IR spectral data (Supplementary file2); Video S1 (Supplementary file3).

Supplementary file1 (PDF 607 KB)

Supplementary file2 (XLSX 1851 KB)

Supplementary file3 (MP4 4303 KB)

## Data Availability

All data relevant to this study are available in this paper and in Supplementary information.
