# Supplementary material for: Physicochemical and biological ageing processes of (micro)plastics in the environment: a multi-tiered study on polyethylene
Source: Environ Sci Pollut Res Int. 2022 Aug 22;30(3):6298–312. doi: 10.1007/s11356-022-22599-4 (PMC9895034; doi:10.1007/s11356-022-22599-4)
Supplement: Supplementary file 1 — Supplementary file1 (PDF 607 KB) [file 11356_2022_22599_MOESM1_ESM.pdf]

Physicochemical and biological ageing processes of (micro)plastics in the environment: a multi-tiered study on polyethylene

Gilberto Binda[1,2*], Giorgio Zanetti[2], Arianna Bellasi[2], Davide Spanu[2], Ginevra Boldrocchi[3], Roberta Bettinetti[3], Andrea Pozzi[2], Luca Nizzetto[1,4*]

[1] Norwegian Institute for Water Research (NIVA), Økernveien 94, 0579 Oslo, Norway

[2] Department of Science and High Technology, University of Insubria, Via Valleggio 11, 22100 Como, Italy

[3] Department of Human and Innovation for the Territory, University of Insubria, Via Valleggio 11, 22100 Como, Italy

[4] RECETOX, Masarik University, Kamenice 753/5, 625 00 Brno, Czech Republic

Supplementary information, Tables S1-S2 and Figs. S1-S4

Table S1 Chemical features of lake water used in the experiments.

Variable	Unit	Value
**Date**	-	18/02/2021
**pH**	-	8.2
**Electrical conductivity**	µS/cm	210
**Alkalinity**	mmol/l	1.5
**Chemical Oxygen Demand**	mg/L	18
**Na^+**	mg/L	2.84
**NH_4^+**	mg/L	0.95
**K^+**	mg/L	1.59
**Mg^{2+}**	mg/L	3.88
**Ca^{2+}**	mg/L	16.75
**F^-**	mg/L	1.3
**Cl^-**	mg/L	3.73
**NO_3^-**	mg/L	3.55
**SO_4^{2-}**	mg/L	15.7

Table S2 Chemical composition of salt depositions in biofouled plastics after UV ageing in air, analyzed by means of SEM-EDX. Data are shown as average ± standard deviation after 5 measurements.

Element	Concentration (wt%)
C	47.81±18.55
O	30.1±10.44
Ca	21.41±1.58
Cl	7.2±4.69
K	4.36±4.84
Na	2.61±1.01

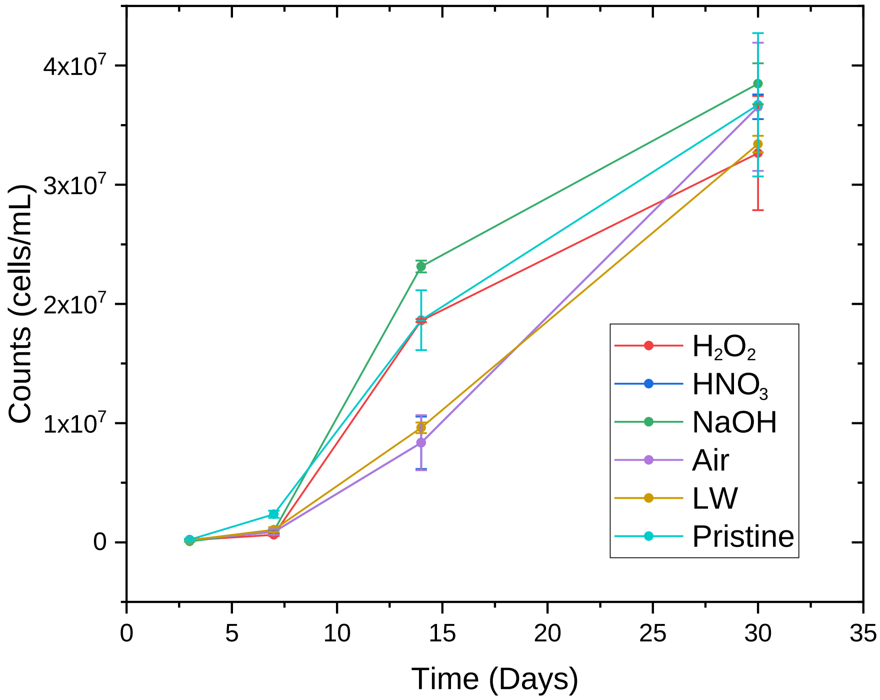

Fig. S1 Algal growth in all the incubation batches for pristine PE fragments (in light blue), and previously UV aged in H_2O_2 (in red), HNO_3 (in blue), NaOH (in green), air (in purple), and lake water (LW, in brown). Error bars indicate the standard deviation values after 3 measurements.

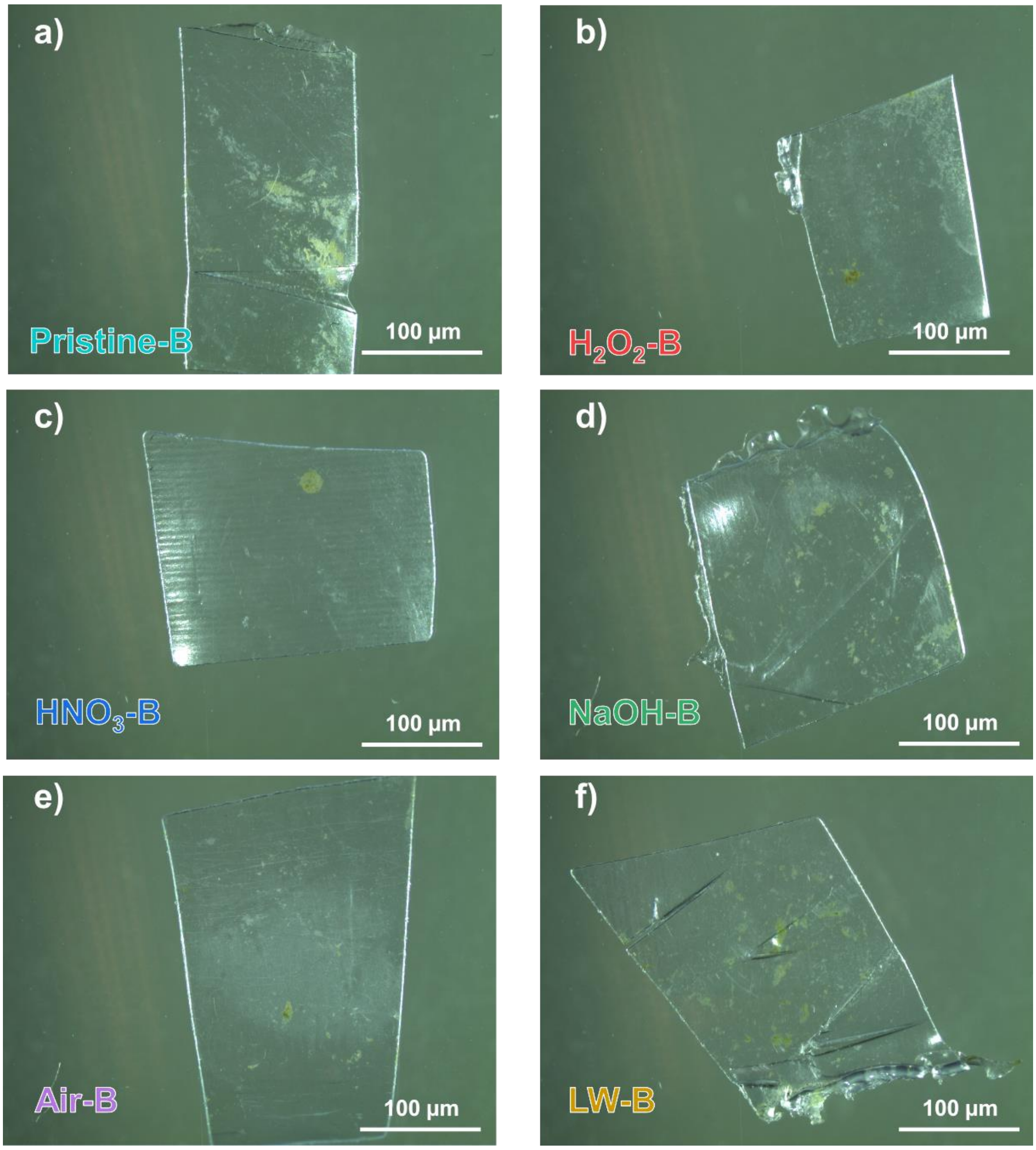

Fig. S2 Stereo microscope images of different PE fragments after 30 days of biofouling. a) pristine PE (Pristine-B), and PE previously UV aged in: b) H_2O_2 (H_2O_2-B), c) HNO_3 (HNO_3-B), d) NaOH (NaOH -B), e) air (Air-B), and f) lake water (LW-B).

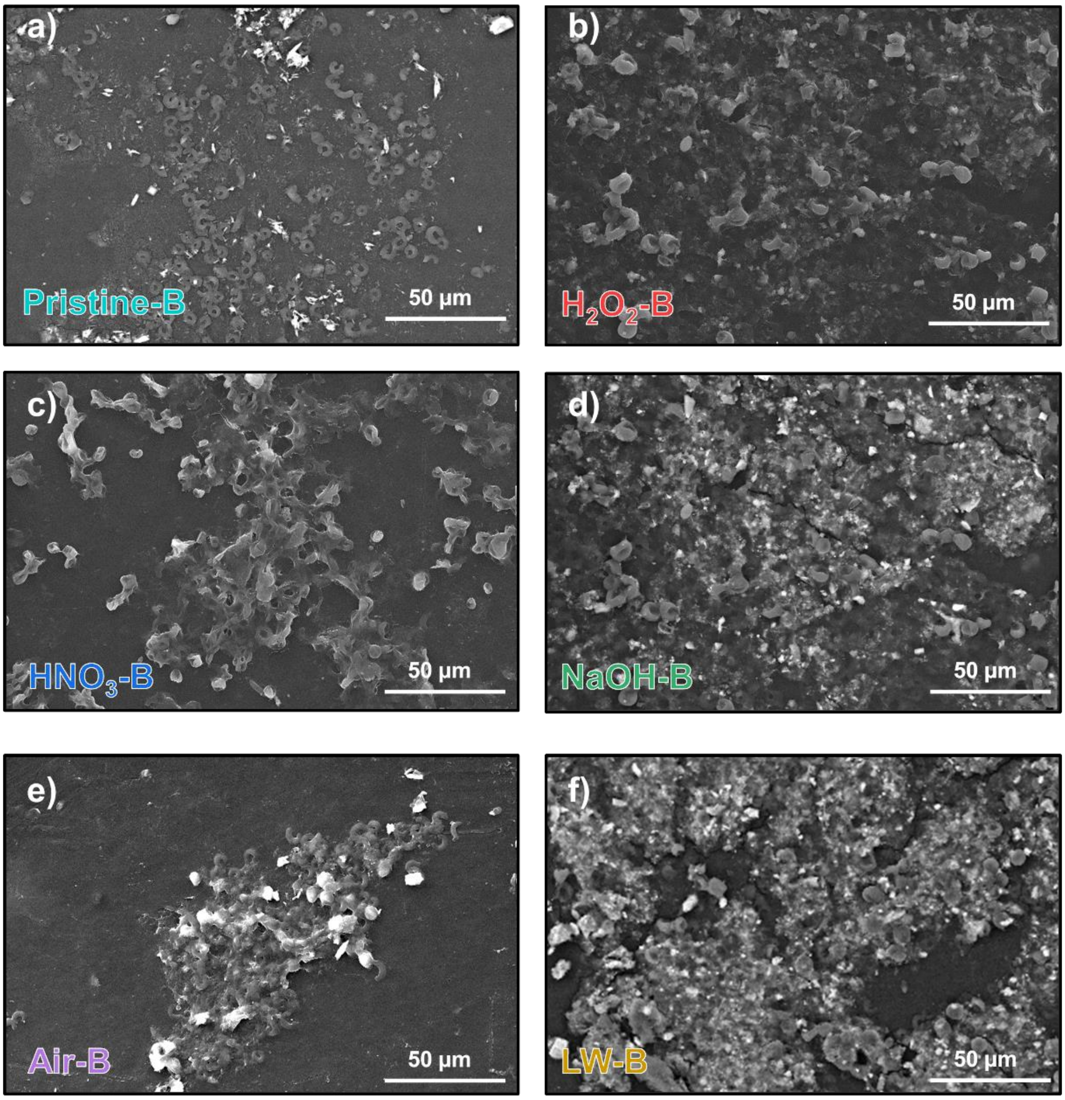

Fig. S3 SEM micrographs of the different PE specimens at 500x magnifications after 30 days of biofouling. a) pristine PE (Pristine-B), and PE previously UV aged in: b) H_2O_2 (H_2O_2-B), c) HNO_3 (HNO_3-B), d) NaOH (NaOH -B), e) air (Air-B), and f) lake water (LW-B).

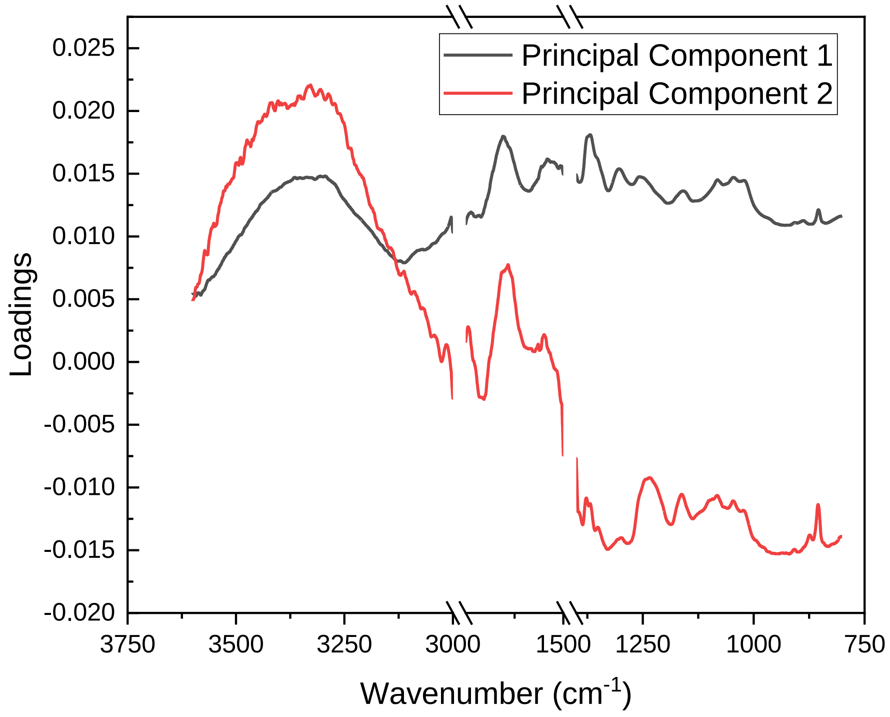

Fig. S4 Loading values at different FT-IR bands for principal component 1 (in black) and 2 (in red), representing the 96.91% and 1.99% of the total variance, respectively.
